# Ultrasound-guided lumbar plexus block versus transversus abdominis plane block for analgesia in children with hip dislocation: A double-blind, randomized trial

**DOI:** 10.1515/med-2022-0581

**Published:** 2022-10-21

**Authors:** Ke Sun, Mei Jin, Xiaoguang Zhang

**Affiliations:** Department of Anesthesiology, Jishuitan Hospital, Beijing, 100035, China

**Keywords:** transversus abdominis, ultrasound, analgesia, hip joint, pediatric

## Abstract

Lumbar plexus block is often used as analgesia for adult hip surgery, but it is rarely used in pediatric patients. This study aimed to compare the efficacy and feasibility of ultrasound-guided lumbar plexus block versus transversus abdominis plane block for postoperative analgesia in children with hip dislocation. Eighty children undergoing unilateral hip dislocation surgeries at our hospital from October 2019 to February 2021 were randomized to the lumbar plexus block group (group L) and transversus abdominis plane block group (group T). Compared with group L, the regional block time in group T was lower (8.0 ± 2.5 vs 11.5 ± 2.3 min, *P* < 0.05), and the ultrasound image definition was better (*P* < 0.05). There were no significant differences in mean blood pressure and heart rate within 24 h (all *P* > 0.05). Children’s Hospital of Eastern Ontario Pain Scale scores were lower in group L than in group T at 18–24 h only (all *P* < 0.05). The satisfying analgesia rate in group L was higher than in group T (87.5 vs 65%, *P* < 0.05). No regional block-related complications were found in both groups. Ultrasound-guided lumbar plexus block showed a longer postoperative analgesic effect in children with hip dislocation compared with transversus abdominis plane block.

## 1 Introduction

### What is known

Transversus abdominis plane block for analgesia after adult hip surgery can reduce the dosage of opioids.

The application of lumbar plexus block or transverse abdominis plane block in children has become more popular.

### What is new

Ultrasound-guided lumbar plexus block has a longer postoperative analgesic effect in children with hip dislocation than transversus abdominis plane block. It provides a convenient anesthesia method for such children.

Developmental dysplasia of the hip (DDH) refers to a spectrum of developmental hip abnormalities ranging from a mildly dysplastic acetabulum and concentrically located femoral head to a severely dysplastic acetabulum and dislocated or dislocatable femoral head [1,2,3]. Although hip instability may affect 1–3% of newborns, DDH is only diagnosed in 0.1–0.5% overall [1,2,4]. It is 4–8 times more common in females [1,5]. The risk factors include breech presentation, oligohydramnios, female gender, and a family history of the condition. DDH is also seen more frequently in the presence of other musculoskeletal anomalies and underlying genetic syndromes [2,3,4,5,6,7].

Most children with DDH require surgical treatment such as pelvic and femoral osteotomy to achieve a concentric reduction of the hip [3,8,9]. Due to the long operation time and significant trauma, the children usually have severe postoperative pain, accompanied by severe stress reactions, leading to poor postoperative recovery outcomes [9].

Lumbar plexus block is often applied as analgesia for adult hip surgery and has a positive effect [10,11], but due to the necessity of using a neurostimulator, it was rarely used in pediatrics before. In recent years, with the development of ultrasound guidance technology, the accuracy of lumbar plexus block in children has been improved, complications can be effectively avoided [12,13,14,15], and its clinical application value has been recognized. Transversus abdominis plane block has been applied in children early, but it was mostly applied in surgeries in the groin area and abdominal wall [16,17]. Transversus abdominis plane block for analgesia after adult hip surgery can reduce the dosage of opioids [18], but its effect in pediatric orthopedics is yet to be verified. In recent years, based on the safety and technical advantages of an ultrasound-guided regional block, the application of lumbar plexus block or transverse abdominis plane block in children has become more popular [19].

Therefore, this trial aimed to compare the analgesic effect and feasibility of ultrasound-guided lumbar plexus block and transversus abdominis plane block in the early postoperative period of pediatric hip dislocation surgery.

## 2 Materials and methods

### 2.1 Study design

This study was a double-blind (evaluators and guardians of children) randomized parallel controlled pilot study. The children were equally divided into two groups receiving different anesthesia methods using the random number table method (without blocks), namely the lumbar plexus block group (group L) and the transverse abdominis plane block group (group T). The randomization sequence was generated by a third-party statistician in opaque, sequentially numbered envelopes. The anesthetist opened the envelopes in order once the patient had entered the operating room. There were no changes to the protocol once the study started.


**Ethics approval:** This work has been carried out in accordance with the Declaration of Helsinki (2000) of the World Medical Association. This study was approved by the Ethics committee of Beijing Jishuitan Hospital (2017-2), and all participants provided written informed consent.

### 2.2 Participants

Child patients who underwent unilateral hip dislocation surgery and pelvic and femoral osteotomy in our hospital from October 2019 to February 2021 were recruited. The scope of surgery included proximal femoral osteotomy and acetabular (pelvic) osteotomy and extensive loosening of soft tissues around the hip joint. The inclusion criteria were (1) 1–6 years of age, (2) child’s height and weight were within ±20% of the standard value (calculation of standard value: child’s body height = age × 7 + 75 cm, weight = age × 2 + 8 kg) [20], (3) American Society of Anesthesiologists (ASA) physical status I or II, (4) children with total dislocation of the hip (degrees I–III) or failed previous closed treatment of acetabular dysplasia or hip subluxation that progressed to total dislocation of the hip and require surgery, (5) unilateral or bilateral hip dislocation, (6) normal nervous system development, and (7) no congenital or genetic diseases apart from DDH. The exclusion criteria were (1) variation, deformity, or local infection of the puncture position; (2) abnormal blood coagulation; (3) central or peripheral nervous system diseases; or (4) any other systemic diseases that can affect surgery or general anesthesia.

### 2.3 Diagnosis standard [8]

The clinical manifestations of DDH include unequal lengths of limbs, limp walking, or swinging gait. Physical examination showed tense adductor muscle, Allis tests positive, and Trendelenburg tests positive. On the pelvic front view, the caput femoris is in the upper outer or lower outer quadrant of the Pekin square, the acetabulum is shallow, and a false bulge is formed.

### 2.4 Anesthesia

The children were fasted and water-deprived for 6 h before the operation. Then, 0.01 mg/kg of atropine was injected intramuscularly 30 min before the operation. Before entering the operation room, 2 mg/kg of ketamine could be injected intramuscularly as basic anesthesia based on the child’s cooperation degree. After entering the operation room, venous access was opened, and ECG, heart rate (HR), non-invasive arterial pressure, pulse oxygen saturation, and respiratory rate were continuously monitored. Continuous mask oxygen inhalation at 2–3 L/min was given. General anesthesia was induced with 0.08 mg/kg of midazolam, 2 μg/kg of fentanyl, 0.1 mg/kg of vecuronium, and 3 mg/kg of propofol. After induction, endotracheal intubation was performed, with volume-controlled ventilation, tidal volume of 8–10 mL/kg, and respiratory rate of 14–18 times/min, which could be adjusted according to the amount of carbon dioxide in exhaled air (ETCO_2_) during the operation. The child was placed in the knee-chest side-lying position, with the surgical side on top.

**Figure 1:**
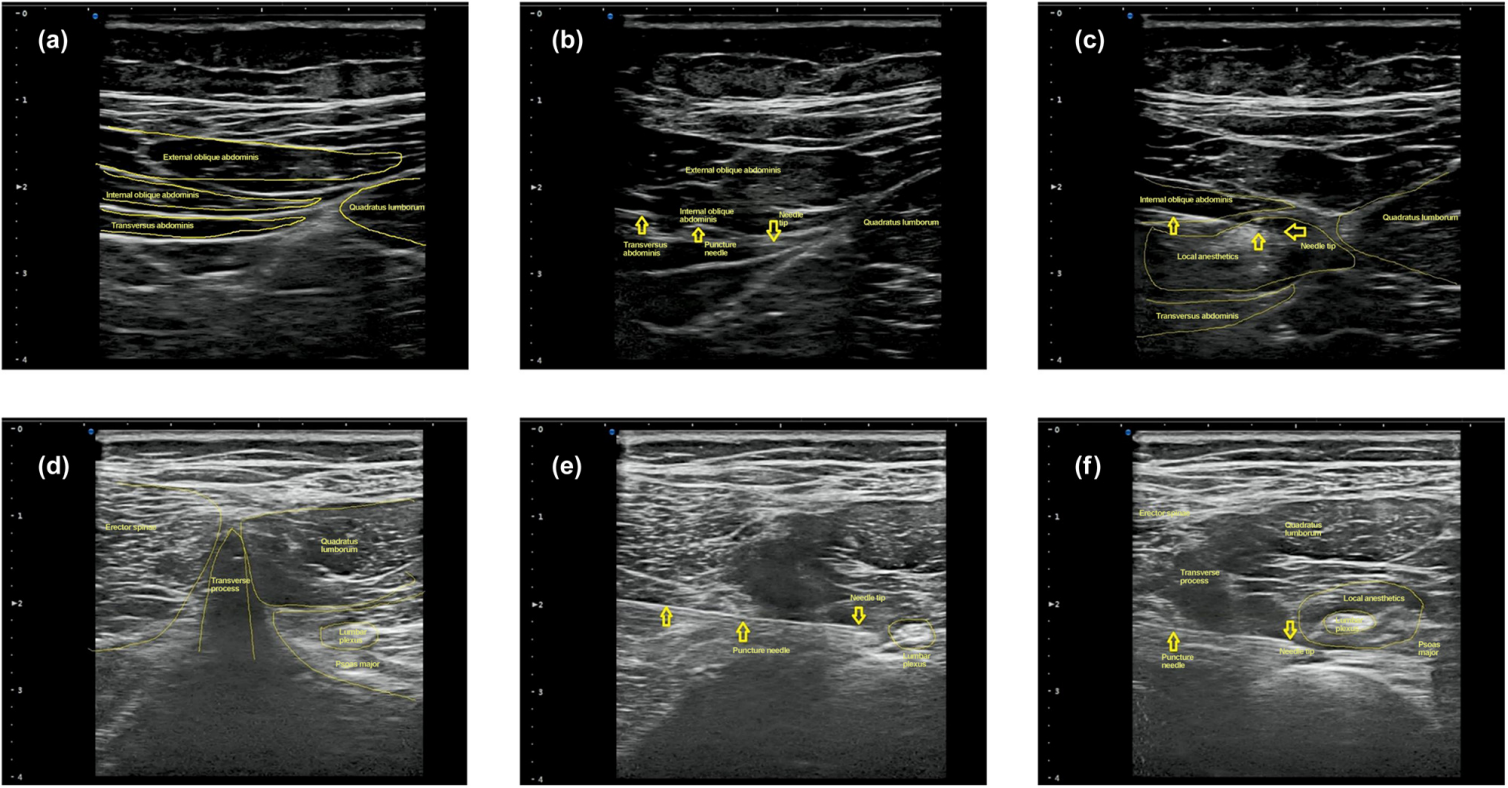
Ultrasound images during local anesthesia. (a–c) Transversus abdominis plane block group (group T). (d–f) Lumbar plexus block group (group L). a/d, b/e, and c/f are representative images for positioning, puncture, and 3 min after drug injection, respectively. The yellow arrows show the position of puncture needles.

Ultrasound guidance was performed using the neuroimaging mode of the M-Turbo ultrasound system (SonoSite, Fujifilm, Bothell, WA, USA), with the transducer connected to the HFL38x/13-6 MHz linear array probe. In group L, the probe was placed above the mid-axillary line iliac crest to identify the shamrock-shaped image composed of the lumbar transverse process, psoas major, erector spinae, and quadratus lumborum, and the position of the L_3_ nerve root was confirmed. The puncture point was located by Winnie’s method, and a local anesthetic was injected into the L_3–4_ gap with an in-plane needle. In group T, the probe was placed between the mid-axillary iliac crest and the costal margin to identify the structure of the musculus obliquus externus abdominis, musculus obliquus internus abdominis, and musculus transversus abdominis. The probe was moved backward until the quadratus lumborum was on the superficial surface of the musculus transversus abdominis. The local anesthetic was injected into between the lateral abdominal wall muscles and the quadratus lumborum muscles with an in-plane needle (Figure 1).

Block was performed in the two groups by anesthesiologists with >3 years of ultrasound experience, who strictly abided by the principle of sterility. The puncture needle was a 22 G, 80 mm nerve stimulation needle (Stimuplex D Plus, B. Braun, Melsungen, Germany), and the local anesthetic was 0.3% ropivacaine hydrochloride (batch number: NBFD, AstraZeneca, London, UK) at 1 mL/kg. A 1- to 2-mL test dose was given before injection to confirm the needle tip position, and withdrawals were repeated during the injection to confirm that blood vessels or the peritoneum had not been punctured by mistake. Surgery started 30 min after the regional block. During the operation, narcosis was maintained with sevoflurane and propofol. The inhalation concentration of sevoflurane was 1–2%, and propofol was infused continuously at 3–5 mg/kg/h. After the operation, the children were moved to the postanesthesia care unit and extubated when becoming awake.

The postoperative evaluation was completed by another anesthesiologist with the same seniority who was not involved in the block.

### 2.5 Osteotomy

After general anesthesia, the child was placed in the supine position, and routine orthopedic disinfection was performed. Open reduction of the hip joint was performed using an anterolateral S-P or Bikini incision. After full exposure and loosening, a T-shaped cut was made to open the joint capsule and clear the contents of the acetabulum. The caput femoris was incorporated into the true acetabulum to achieve a concentric hip after reduction, and a V-shaped posterior capsulorrhaphy was performed. After concentric circle reduction was achieved, pelvic osteotomy was performed to reconstruct the acetabulum. For those who need a change of direction of the acetabulum, a triple osteotomy or Salter osteotomy was performed. For those who need a change of form of the acetabulum, Pemberton osteotomy was performed. The aforementioned steps were performed with the same surgical incision (S-P or Bikini). The surgical field was in the groin area, including the caput femoris, acetabulum, surrounding soft tissues, and pelvis. Osteotomy was performed on the proximal femur, with internal fixation with a steel plate, and an incision was made on the outside of the thigh. Proximal femur shortening osteotomy was performed to reduce the pressure on the acetabular head (caput femoris and acetabulum), or varus rotational osteotomy was performed to correct an excessive anteversion angle and cervical shaft angle (collum femoris and femoral shaft). The surgical field was in the proximal femur and surrounding soft tissues.

### 2.6 Postoperative cares

General care: After returning to the ward, the children were not fully awake. They laid down with their head tilted to one side. Continuous low-flow oxygen inhalation (3 L/min) was maintained. Body temperature, HR, non-invasive blood pressure, and respiratory rate were monitored. The color of the limb end, skin temperature, sensation, and movement were observed. Whether there was blood oozing at the incision was observed. Sleep and bowel movements were recorded.

Orthopedic care: After the operation, the child was plastered or fixed with limb support to keep the affected limb in position and avoid flexing the affected limb. The part under pressure was observed to avoid pressure sores.

### 2.7 Data collection and observation indicators

There were no changes to the outcomes after the trial started. The baseline data and clinical indicators of the two groups of children were recorded, including ASA grade, operation time, and regional block time. The mean blood pressure (MBP), HR, and Children’s Hospital of Eastern Ontario Pain Scale (CHEOPS) scores [21] of the children were recorded at 2, 6, 12, 18, and 24 h after surgery. The CHEOPS pain score uses six behavioral indicators: crying, facial expressions, speech, leg activity, physical activity, and wound touchability. Each item is defined as 0–2 points or 1–3 points, and the total score is 4–13 points. CHEOPS pain scores <6 were defined as analgesic satisfied, and the satisfying analgesia rate was calculated.

The regional block time was defined as the time taken from the start of the ultrasound scan to the completion of the local anesthetic injection. The ultrasound images were evaluated with a 4-point method [22]: 0 points, unable to display; 1 point, the anatomical structure and injection target position are not clear, and the puncture needle is partially developed; 2 points, the anatomical structure, and injection target position are clear, but the puncture needle tip is poorly developed, and local anesthetic diffusion is limited; 3 points, typical anatomical structure, and injection target position, the puncture needle tip can be accurately identified, and local anesthetic diffusion is complete. Images with ≥2 points meant that local anesthetics could be injected.

Whether there were local anesthetic toxic reactions, bleeding, or hematoma at the puncture position; whether there was a bilateral block or kidney injury in group L; and whether there were complications such as peritoneal puncture and intestinal injury in group T were observed.

### 2.8 Statistical analysis

As it was an exploratory study, no sample size was calculated. All children matching the eligibility criteria during the study period were asked to participate. SPSS 16.0 (SPSS, Chicago, IL, USA) was used for data analysis. The continuous data were tested for normal distribution using the Wilk–Shapiro test. Those conforming to the normal distribution were presented as mean ± standard deviation and were analyzed using Student’s *t*-test (between-group comparisons) and two-way ANOVA with the *post hoc* Student-Newman-Keuls test (for within-group comparisons among time points). Data that were not in a normal distribution were analyzed using the non-parametric Friedman test, and if the test results were statistically significant, the *P*-value was further adjusted using the Bonferroni method, and then, multiple comparisons were made using the Wilcoxon matched-pairs signed-rank test. Categorical data were expressed as *n* (%) and analyzed using the chi-square test or Fisher’s exact test. *P*-Values <0.05 were considered statistically significant.

## 3 Results

### 3.1 Baseline characteristics of the participants

From October 2019 to February 2021, 80 child patients who underwent unilateral hip dislocation and pelvic osteotomy and orthopedic surgeries were enrolled, aged 1–6 years, ASA physical status I or II, and weighting 8–25 kg. There were no significant differences in age, sex composition, height, weight, ASA grading, and operation time between the two groups (all *P* > 0.05; Table 1).

**Table 1:** Characteristics of the participants

	L group (*n* = 40)	T group (*n* = 40)
Age (years)	3.0 ± 1.2	3.2 ± 1.3
Sex *n* (%)		
Male	7 (17.5)	5 (12.5)
Female	33 (82.5)	35 (87.5)
Height (cm)	89.6 ± 12.5	87.5 ± 14.2
Weight (kg)	14.9 ± 3.5	15.0 ± 3.5
ASA grade, *n* (%)		
I	39 (97.5)	38 (95)
II	1 (2.5)	2 (5)
Operation time (min)	188.0 ± 23.0	186.5 ± 27.2

The data are displayed using mean ± standard deviation or *n* (%).

Group L: lumbar plexus block group; Group T: transverse abdominal muscle plane block group; ASA: American Society of Anesthesiologists.

### 3.2 Regional block and pain

All patients completed the regional block. Compared with group L, group T had a lower regional block time (8.0 ± 2.5 vs 11.5 ± 2.3 min, *P* < 0.05), and ultrasound image clarity was better (*P* < 0.05; Table 2). MBP and HR within 24 h after operation between the two groups were compared (Tables A1 and A2), and the differences were not statistically significant (all *P* > 0.05).

**Table 2:** Comparison of the regional block between the two groups

	L group (*n* = 40)	T group (*n* = 40)
Regional blocking time (min)	11.5 ± 2.3	8.0 ± 2.5^a^
Ultrasound image scoring		
2 Points	9 (22.5)	2 (5)^a^
3 Points	31 (77.5)	38 (95)^a^

^a^ *P* < 0.05 vs group L.

The data are displayed using mean ± standard deviation (SD) or *n* (%).

Group L: lumbar plexus block group; Group T: transverse abdominal muscle plane block group.

Anesthesia injection was performed when the ultrasound image score was ≥2 points.

There were no significant differences in the CHEOPS pain scores within 12 h after surgery between the two groups (all *P* > 0.05). Group L had lower scores than group T at 18–24 h (T_18_: 5 (4, 8) vs 5 (4.9), *P* = 0.01; T_24_: 5 (4, 9) vs 5 (4, 9), *P* = 0.01). Pain scores were significantly higher in the T group at 18 and 24 h postoperatively compared to 2 h postoperatively (T_2_ vs T_18_: 5 (4,6) vs 5 (4,9), *P* < 0.01; T_2_ vs T_24_: 5 (4,6) vs 5 (4,9), *P* < 0.01), but no significant differences were found in the L group (all *P* > 0.05). The satisfying analgesia rate of group L was higher than that of group T (87.5% vs 65%, *P* < 0.05; Table 3).

**Table 3:** Comparison of the analgesic effects in the two groups within 24 h after surgery

	L group (*n* = 40)	T group (*n* = 40)
CHEOPS pain score		
T_2_	5 (4,6)	5 (4,6)
T_6_	5 (4,6)	5 (4,6)
T_12_	5 (4,6)	5 (4,6)
T_18_	5 (4,8)	5 (4,9)^a,b^
T_24_	5 (4,9)	5 (4,9)^a,b^
Satisfying analgesia rate (%)	87.5	65^a^

The data are displayed using median (range).

^a^ *P* < 0.05 vs group L; ^b^ *P* < 0.05 vs T_2_, using Friedman test.

Group L: lumbar plexus block group; Group T: transverse abdominal muscle plane block group; CHEOPS: Children’s Hospital of Eastern Ontario Pain Scale; T_2_, T_6_, T_12_, T_18_, and T_24_ represent postoperative 2, 6, 12, 18, and 24 h.

### 3.3 Safety

There were no local anesthetic toxic reactions, bleeding or hematoma at the puncture position, or complications related to regional block in the two groups.

## 4 Discussion

This trial aimed to compare the effect and feasibility of ultrasound-guided lumbar plexus block and transversus abdominis plane block for early postoperative analgesia in children with hip dislocation surgery. The results suggest that ultrasound-guided lumbar plexus block has a longer postoperative analgesic effect in children with hip dislocation than transversus abdominis plane block.

During hip surgery, a part of the abdominal wall and inguinal ligaments are separated from the iliac crest and reattached before closing the wound. Part of the postoperative pain is due to this soft tissue manipulation and the aponeurosis pulling on the iliac crest. Transverse abdominal block effectively anesthetizes the entire surgical area, including the incision, iliac crest, and groin. At the same time, local anesthetics can temporarily paralyze the abdominal muscles and reduce the tension of the abdominal wall on the iliac crest, thus reducing the degree of postoperative pain and reducing the need for opioids [18]. The pain stimulation caused by hip surgery in children is often stronger and lasts longer than in adults, and even causes long-term adverse effects, such as reduced pain tolerance, conversion to chronic pain, fear of reoperation, and psychological or behavioral changes [23]. A regional block is an important analgesic method in the perioperative period of rapid rehabilitation surgery. It can reduce the dosage of opioids after surgery and lower the incidence of adverse events, but it also has a regulatory effect on the body’s immune function and avoids excessive stress reactions [12,13,14,15,16,17]. Ultrasound-guidance technology has improved the safety and effectiveness of regional blocks in children, enabling the implementation of lumbar plexus block or transverse abdominis plane block, which was difficult to perform on children in the past. Still, the operation depends on factors such as the experience of the anesthesiologist, ultrasound equipment, and puncture needles; thus, the clinical applications are still limited. Ropivacaine is a long-acting local anesthetic of amide-type [24]. Increasing the dose can prolong the time of regional block analgesia, but the risks of toxic reaction and nerve damage also increase accordingly [24]. The 0.3% ropivacaine 1-mL/kg dose used in this experiment is currently the most commonly used dose for pediatric regional block in China, and it is within the range of pediatric safe medication [25].

Ultrasound-guided transversus abdominis plane block has a variety of needle access methods. According to the block range, the subcostal method, lateral approach, or posterior approach can be selected [26]. The advantages of the posterior approach include a long-acting time and wide block range [26,27]. Lochel et al. [18] found that posterior transversus abdominis plane block can be used for early analgesia in adult hip surgery. After the local anesthetic is injected into the potential fascial space, a part of the local anesthetic directly acts on the anterior branch of the spinal nerve. There are extensive communicating branches between the nerves in the transverse abdominis plane to form the musculus transversus abdominis nerve plexus so that a single injection can block multiple segments [26,27]. The rest of the local anesthetic reaches the paravertebral space through the quadratus lumborum muscle and expands the range of the block [26,27]. Children have loose tissues, and thus, local anesthetic diffuses more easily than in adults. It has been reported that with a larger dose of local anesthetics, even the femoral nerve can be blocked [26]. The block of nervus iliohypogastricus from T_12_ and L_1_ and nervus ilioinguinalis from L_1_ has an analgesic effect on pediatric hip surgery [26].

Compared with transversus abdominis plane block, ultrasound-guided lumbar plexus block with the “shamrock” method has been widely used for analgesia of adult hip and lower extremity surgeries [28,29]. A local anesthetic is usually injected at the L_3_ level. The blocking range can reach the innervation area of T_12_–L_4_. With its diffusion downward, part of the sacral plexus nerve can also be blocked [30]. Under ultrasound guidance, a local anesthetic can be accurately injected around the lumbar plexus. When the diffusion is good, the “donut sign” can be seen in the image, and the local anesthetic is in full contact with the nerve fiber to ensure the best blocking effect [19]. It can explain the longer analgesia time and more complete effect in group L than in group T.

Children have poor expression and communication skills, and their pain tolerance performance is also different from that of adults [31,32]. Behavioral scores are often used to evaluate analgesic effects [31,32]. The children in this trial had lower CHEOPS pain scores. In addition to the regional block to reduce the pain, the application of external fixation with a cast after the operation and the immobilization of the surgical site also reduced the pain caused by movement.

In this trial, a high-frequency linear array probe was selected for scanning, which can identify the echo difference more precisely. The nerve stimulation needle used was coated with high molecular polymer on the surface. Its porous microstructure can lock air microbubbles and enhance the reflection effect of ultrasound, facilitating the development of the needle tip. The anatomical structure of children is shallower than that of adults, the muscle thickness is not large, and the distance from the skin to the peritoneum is about 1–2 cm. However, the nerve fibers of the lumbar plexus in children are slender and difficult to distinguish from the psoas major muscle. Deep needle insertion may cause damage to the vertebral artery and bilateral blockage, which would introduce a large dosage of local anesthetics into the blood vessel or spinal canal, causing serious consequences [33,34]. Therefore, the unclear ultrasound images of some children in group L resulted in a longer block time. When the transversus abdominis plane is blocked, the injection target position is far from the nerves and blood vessels, and the peritoneum is highly recognizable under ultrasound. Thus, it is safer and more convenient.

Compared with adults, the anatomical structure of children is more delicate. The lumbar plexus is in the deep tissues, and the surrounding anatomical structure is complex, but the nerves, blood vessels, muscles, and bones have completely different acoustic characteristics. Ultrasound can provide clear imaging of the tissue structure of the local cross-section due to the different echoes. In children, because the subcutaneous tissue and muscle layers are thinner than in adults and the lumbar plexus is closer to the body surface, a high-frequency probe can be selected to improve the recognition of the lumbar plexus and surrounding tissues to timely guide the needle to reach the desired position. In addition, local anesthetics diffusion can be observed in real-time, allowing the dynamic fine adjustment of the tip position, improving the accuracy of injection, avoiding retroperitoneal hematoma due to a too deep puncture, kidney damage, or diffusion of the anesthetics in the spinal canal, thereby improving the safety of the procedure.

This study has limitations. Because children are not good at expression and communication, and the performance of pain tolerance is different from adults, behavioral indicators are usually used to evaluate the effect of postoperative analgesia in children. Although the five indicators used by CHEOPS are all objective behaviors, they depend on the subjective judgment of the rater [21]. In order to reduce interference, parents are required to guide the children not to focus on the surgical site. The rater was blind to the grouping of the children and had to try to avoid whitecoats, which caused fear in children during the follow-up visits. In addition, this trial was performed at a single center, and the sample size was small. There are subjective factors in the judgment of ultrasound images and postoperative scoring which interfere with the experimental results.

In summary, compared with transverse abdominis plane block, ultrasound-guided lumbar plexus block is better for early analgesia after hip dislocation surgery in children, with a longer analgesic time. Ultrasound-guided transversus abdominis plane block is relatively easy to operate and can be used as an alternative to lumbar plexus block. Future studies should explore different strategies for blocking, different drugs and dosages, and in different age groups in order to improve patient outcomes.

## Abbreviations

CHEOPS: Children’s Hospital of Eastern Ontario Pain Scale
DDH: developmental dysplasia of the hip
HR: heart rate
MBP: mean blood pressure

## Appendix

**Table A1:** Comparison of MBP between the two groups within 24 hours after surgery

	L group (*n* = 40)	T group (*n* = 40)
MBP (mmHg)		
T_2_	63.0 ± 5.8	64.2 ± 5.5
T_6_	61.3 ± 5.9	63.5 ± 4.4
T_12_	62.1 ± 5.9	64.3 ± 4.3
T_18_	65.4 ± 5.7	66.2 ± 4.8
T_24_	65.5 ± 5.2	66.8 ± 4.6

The data is displayed using mean ± standard deviation (SD).

MBP: mean arterial pressure; Group L: lumbar plexus block group; Group T: transverse abdominal muscle plane block group. T_2_, T_6_, T_12_, T_18,_ and T_24_ represent postoperative 2, 6, 12, 18, and 24 h.

**Table A2:** Comparison of HR in the two groups within 24 h after operation

	L group (*n* = 40)	T group (*n* = 40)
HR (beats/min)		
T_2_	105.8 ± 13.5	107.5 ± 12.9
T_6_	103.7 ± 13.2	107.8 ± 10.5
T_12_	104.6 ± 11.7	107.9 ± 11.9
T_18_	107.3 ± 14.4	112.8 ± 12.8
T_24_	110.1 ± 14.2	115.4 ± 11.7

The data is displayed using mean ± standard deviation (SD).

HR: heart rate; Group L: lumbar plexus block group; Group T: transverse abdominal muscle plane block group. T_2_, T_6_, T_12_, T_18,_ and T_24_ represent postoperative 2, 6, 12, 18, and 24 h.
